# Association of *solute carrier family 30 A8 zinc transporter gene* variations with gestational diabetes mellitus risk in a Chinese population

**DOI:** 10.3389/fendo.2023.1159714

**Published:** 2023-05-31

**Authors:** Qiaoli Zeng, Bing Tan, Fengqiong Han, Xiujuan Huang, Jinzhi Huang, Yue Wei, Runmin Guo

**Affiliations:** ^1^ Department of Internal Medicine, Shunde Women and Children’s Hospital (Maternity and Child Healthcare Hospital of Shunde Foshan), Guangdong Medical University, Foshan, Guangdong, China; ^2^ Key Laboratory of Research in Maternal and Child Medicine and Birth Defects, Guangdong Medical University, Foshan, Guangdong, China; ^3^ Maternal and Child Research Institute, Shunde Women and Children’s Hospital (Maternity and Child Healthcare Hospital of Shunde Foshan), Guangdong Medical University, Foshan, Guangdong, China; ^4^ Department of Endocrinology, Second Affiliated Hospital of Guangdong Medical University, Zhanjiang, China; ^5^ Department of Obstetric, Shunde Women and Children’s Hospital (Maternity and Child Healthcare Hospital of Shunde Foshan), Guangdong Medical University, Foshan, Guangdong, China; ^6^ Department of Children’s Health, Shunde Women and Children’s Hospital (Maternity and Child Healthcare Hospital of Shunde Foshan), Guangdong Medical University, Foshan, Guangdong, China; ^7^ Department of Gynaecology, Shunde Women and Children’s Hospital (Maternity and Child Healthcare Hospital of Shunde Foshan), Guangdong Medical University, Foshan, Guangdong, China; ^8^ Department of Ultrasound, Shunde Women and Children’s Hospital (Maternity and Child Healthcare Hospital of Shunde Foshan), Guangdong Medical University, Foshan, Guangdong, China; ^9^ Department of Endocrinology, Affiliated Hospital of Guangdong Medical University, Zhanjiang, Guangdong, China

**Keywords:** gestational diabetes mellitus, solute carrier family 30 A8 zinc transporter, SNP, rs13266634, rs2466293, case-control study

## Abstract

**Background:**

The *solute carrier family 30 A8 zinc transporter* (*SLC30A8*) plays a crucial role in insulin secretion. This study aimed to investigate the impact of *SLC30A8* gene polymorphisms on gestational diabetes mellitus (GDM).

**Methods:**

The research objective was to select 500 patients with GDM and 502 control subjects. Rs13266634 and rs2466293 were genotyped using the SNPscan™ genotyping assay. Statistical tests, such as the chi-square test, t-test, logistic regression, ANOVA, and meta-analysis, were conducted to determine the differences in genotypes, alleles, and their associations with GDM risk.

**Results:**

Statistically significant differences were observed in age, pregestational BMI, SBP, DBP, and parity between individuals with GDM and healthy subjects (*P* < 0.05). After adjusting for these factors, rs2466293 remained significantly associated with an increased risk of GDM in overall subjects (GG+AG vs. AA: OR = 1.310; 95% CI: 1.005-1.707; *P* = 0.046, GG vs. AA: OR = 1.523; 95% CI: 1.010-2.298; *P* = 0.045 and G vs. A: OR = 1.249; 95% CI: 1.029-1.516; *P* = 0.024). Rs13266634 was still found to be significantly associated with a decreased risk of GDM in individuals aged ≥ 30 years (TT vs. CT+CC: OR = 0.615; 95% CI: 0.392-0.966; *P* = 0.035, TT vs. CC: OR = 0.503; 95% CI: 0.294-0.861; *P* = 0.012 and T vs. C: OR =0.723; 95% CI: 0.557-0.937; *P* = 0.014). Additionally, the haplotype CG was found to be associated with a higher risk of GDM (*P* < 0.05). Furthermore, pregnant women with the CC or CT genotype of rs13266634 exhibited significantly higher mean blood glucose levels than those with the TT genotype (*P* < 0.05). Our findings were further validated by the results of a meta-analysis.

**Conclusion:**

The *SLC30A8* rs2466293 polymorphism was found to be associated with an increased risk of GDM, while rs13266634 was associated with a decreased risk of GDM in individuals aged ≥ 30 years. These findings provide a theoretical basis for GDM testing.

## 1 Introduction

Gestational diabetes mellitus (GDM) is a global concern, and its incidence has increased by over 30% in numerous countries during the past few years (1, 2). GDM is characterized by β-cell dysfunction, insulin resistance, and abnormal glucose utilization (3, 4), but its pathogenesis is not yet clear. Increasing evidence indicates that environmental and genetic factors are implicated in the development of GDM. Single nucleotide polymorphisms (SNPs) are a common type of genetic variation, and polymorphisms in different genes may be associated with GDM (5).

The *solute carrier family 30 A8 zinc transporter* (*SLC30A8*) gene encodes ZnT8, which is primarily expressed in pancreatic β-cells and is in charge of delivering zinc from the cytoplasm into insulin vesicles (6). *SLC30A8* is involved in the secretion of insulin (7). The zinc stabilizes the insulin hexamer in secretory insulin vesicles, making it resistant to degradation (8). Insulin packaged into secretory vesicles can be released immediately upon glucose stimulation (7). The rs13266634 polymorphism is a missense C to T variant in exon 9 of the *SLC30A8* gene, and the amino acid changes from arginine (R) to tryptophan (W) at position 325 (8). Thus, rs13266634 has been thought to be related to diabetes risk, as it affects the expression of *SLC30A8*, and negative regulation of ZnT8 is considered to disrupt the stability of insulin molecules (9). The polymorphism rs2466293 is in the 3′-UTR of the *SLC30A8* gene, and rs2466293 may impact *SLC30A8* post-transcriptional regulation by binding to miRNA (10). MiRNAs are closely related to gene level regulation; hence, rs2466293 in the seed sites of miRNA targets can create or disrupt miRNA-binding sites that further influence disease susceptibility (11). In this context, this study researched the influence of rs13266634 and rs2466293 polymorphisms on GDM risk.

## 2 Materials and methods

### 2.1 Study subjects

From 1 August 1 2021 to 31 January 31 2022, a total of 1,002 unrelated Chinese Han pregnant women (500 GDM cases and 502 controls) were recruited for our study at the obstetric clinic of Shunde Maternal and Child Health Hospital, Guangdong Medical University. All individuals underwent a routine 75-gram oral glucose tolerance test (OGTT) during 24-28 weeks of gestation. A control group consisting of pregnant women at 24 to 28 weeks of gestation was selected over the same period. The inclusion criteria were as follows: voluntarily provided written informed consent, not previously diagnosed with diabetes, Han nationality, aged ≥ 18 years, no pregnancy complications, and not taking hypoglycemic medicines. Participants who did not meet the above criteria were excluded.

### 2.2 Data collection

Information including age, height, pregestational weight, parity (primipara or multipara), blood pressure, race, pregnancy condition, and other clinical information were obtained at 24-28 gestational weeks. Pregestational body mass index (pre-BMI, Kg/m^2^) was calculated as pregestational weight (Kg) divided by height squared (m^2^). The Chinese standards for obesity were as follows: underweight (< 18.5 Kg/m^2^), normal (18.5-24 Kg/m^2^), overweight (24-28 Kg/m^2^), and obese (≥ 28 kg/m^2^).

### 2.3 SNP genotyping

A total of 2 mL of EDTA-treated blood was immediately stored in the freezer. Genomic DNA was extracted and purified from blood cells by a QIAamp DNA Blood Kit (Qiagen, Germany). Genotypes of candidate SNPs were determined using the SNPscan™ genotyping assay (Genesky Technologies Inc., Shanghai, China). Pre-experiments were conducted before formal experiments. In order to check the genotyping data accuracy, 6% of the samples were randomly selected for duplicate analysis using Sanger sequencing.

### 2.4 Statistical analyses

Continuous variables following normal distribution were reported as means ± SD, and the independent sample t-test was used to determine the differences between the relevant parameters of the two groups. In cases where the assumption of normality was violated, non-parametric tests were employed. Qualitative data were analyzed using the chi-square (χ2) test. The Hardy-Weinberg equilibrium (HWE) test, assessed through the goodness-of-fit χ2, was used to ensure that the control group was representative of the population. The risk of GDM was evaluated using six genetic models, namely, codominant homozygous, codominant heterozygous, dominant, recessive, overdominant, and allele models, through the χ2 test and logistic regression analysis. Crude and adjusted odds ratios (ORs) and their corresponding 95% confidence intervals (CIs) were presented, with adjustments made for covariates such as age, pre-BMI, etc. Stratified analysis was performed to further examine the potential influence of age and pre-BMI on the results. The frequency distribution of haplotypes was calculated using Haploview 4.2 software. The association between SNPs and blood glucose levels was investigated using one-way ANOVA. For multiple comparisons, the least significant difference (LSD) method was used. Statistical analyses were performed using SPSS 20.0 (SPSS Inc., Chicago, IL, USA), and a *P*-value < 0.05 was considered statistically significant.

## 3 Results

### 3.1 General clinical characteristics of the subjects

The study included 500 GDM cases and 502 non-diabetic controls for the evaluation of the *SLC30A8* genotype. **Table 1** presents the clinical baseline information and stratified features. The mean age, pre-BMI, systolic blood pressure (SBP), diastolic blood pressure (DBP), fasting plasma glucose (FPG), 1 h-PG, and 2 h-PG were significantly higher in the GDM group than in the control group (*P* < 0.05). Furthermore, there was a significant difference in parity between the GDM and control groups (*P* < 0.05).

**Table 1:** Basic and stratified characteristic of participants of the study.

Variables	Cases (%)	Controls (%)	t/χ2	*P*
	(n = 500)	(n = 502)		
Age, year (mean ± SD)	31±4	29±4	-8.56	**< 0.001**
pre-BMI, kg/m^2^	21.51±3.10	20.53±2.58	-5.42	**< 0.001**
SBP, mmHg	117±11	114±10	-3.53	**< 0.001**
DBP, mmHg	70±8	68±7	-3.23	**0.001**
FPG, mmol/L	4.82±0.64	4.50±0.31	-9.75	**< 0.001**
1h-PG, mmol/L	10.17±1.60	7.66±1.27	-26.22	**< 0.001**
2h-PG, mmol/L	8.91±1.60	6.69±0.99	-25.85	**< 0.001**
Parity (n)			8.88	**0.003**
Primipara	210 (42)	258 (51.4)		
Multipara	290 (58)	244 (48.6)		
**Variables**	Cases (%)	Controls (%)	**χ2**	***P***
	(n = 500)	(n = 502)		
Age, year			49.2	**< 0.001**
< 30	192 (38.4)	304 (60.6)		
≥ 30	308 (61.6)	198 (39.4)		
pre-BMI, kg/m^2^			27.8	**< 0.001**
< 18.5	67 (13.4)	95 (18.9)		
18.5 ≤ BMI < 24	336 (67.2)	365 (72.7)		
≥ 24	97 (19.4)	42 (8.3)		

pre-BMI pre-gestational body mass index, SBP systolic blood pressure, DBP diastolic blood pressure, FPG fasting plasma glucose, bold values indicate the P ≤ 0.05.

### 3.2 The association between polymorphisms and GDM risk

#### 3.2.1 Overall analysis results

**Table 2** presents the minor allele frequency (MAF) and the results of the HWE analysis for two SNPs in the control group. The results were in conformity with HWE (*P* > 0.05). **Table 3** shows the ORs with corresponding 95% CIs and associated *P* values estimated for the relationship between genotypes and GDM in the six models (codominant homozygous, codominant heterozygous, dominant, recessive, overdominant, and allele models) for each polymorphism. *SLC30A8* rs2466293 was found to be significantly associated with an increased risk of GDM in the dominant model (GG+AG vs. AA: OR = 1.288; 95% CI: 1.003-1.655; *P* = 0.047), codominant homozygous model (GG vs. AA: OR = 1.499; 95% CI: 1.014-2.217; *P* = 0.043), and allele model (G vs. A: OR = 1.237; 95% CI: 1.029-1.487; *P* = 0.023). Further evaluation was performed using a logistic regression method to adjust for age, pre-BMI, SBP, DBP, and parity. The results indicated a strong association between *SLC30A8* rs2466293 and an increased risk of GDM in the dominant model (GG+AG vs. AA: OR = 1.310; 95% CI: 1.005-1.707; *P* = 0.046), codominant homozygous model (GG vs. AA: OR = 1.523; 95% CI: 1.010-2.298; *P* = 0.045), and allele model (G vs. A: OR = 1.249; 95% CI: 1.029-1.516; *P* = 0.024). However, no significant association was found in rs13266634.

**Table 2:** SNPs information and HWE test in the controls.

SNP	Min/Maj	Chr. position	MAF	HWE (*P*)
rs13266634	T/C	chr8:117172544	0.473	0.894
rs2466293	G/A	chr8:117173699	0.325	0.627

Min minor allele, Maj major allele, MAF frequency of minor allele, HWE Hardy–Weinberg equilibrium.

**Table 3:** The associations between SNPs in SLA30C8 gene and GDM risk in overall subjects.

Model	Cases (%)	Controls (%)	Crude OR	Crude *P*	Adjusted OR	Adjusted *P*
	(n = 500)	(n = 502)	(95 % CI)		(95 % CI)	
rs13266634						
Codominant model						
CC	161 (32.2)	142 (28.3)	1(ref)		1(ref)	
CT	240 (48.0)	245 (48.8)	0.864 (0.648-1.152)	0.319	0.824 (0.609-1.116)	0.212
TT	99 (19.8)	115 (22.9)	0.759 (0.535-1.078)	0.124	0.754 (0.520-1.092)	0.135
Aelle model						
C	562 (56.2)	529 (52.7)	1(ref)		1(ref)	
T	438 (43.8)	475 (47.3)	0.868 (0.728-1.035)	0.115	0.862 (0.716-1.037)	0.115
Dominant Model						
CC	161 (32.2)	142 (28.3)	1(ref)		1(ref)	
TT+CT	339 (67.8)	360 (71.7)	0.831 (0.634-1.008)	0.178	0.802 (0.604-1.006)	0.129
Recessive Model						
CT+CC	401 (80.2)	387 (77.1)	1(ref)		1(ref)	
TT	99 (19.8)	115 (22.9)	0.831 (0.614-1.125)	0.23	0.848 (0.616-1.169)	0.314
Overdominant model						
TT+CC	260 (52.0)	257 (51.2)	1(ref)		1(ref)	
CT	240 (48.0)	245 (48.8)	0.968 (0.756-1.241)	0.799	0.926 (0.713-1.203)	0.565
rs2466293						
Codominant model						
AA	201 (40.2)	233 (46.4)	1(ref)		1(ref)	
AG	224 (44.8)	211 (42.0)	1.231 (0.943-1.606)	0.127	1.251 (0.944-1.658)	0.119
GG	75 (15.0)	58 (11.6)	1.499 (1.014-2.217)	**0.043**	1.523 (1.010-2.298)	**0.045**
Aelle model						
A	626 (62.6)	677 (67.4)	1(ref)		1(ref)	
G	374 (37.4)	327 (32.6)	1.237 (1.029-1.487)	**0.023**	1.249 (1.029-1.516)	**0.024**
Dominant Model						
AA	201 (40.2)	233 (46.4)	1(ref)		1(ref)	
GG+AG	299 (59.8)	269 (53.6)	1.288 (1.003-1.655)	**0.047**	1.310 (1.005-1.707)	**0.046**
Recessive Model						
AG+AA	425 (85.0)	444 (88.4)	1(ref)		1(ref)	
GG	75 (15.0)	58 (11.6)	1.351 (0.935-1.951)	0.109	1.360 (0.925-1.999)	0.118
Overdominant model						
GG+AA	276 (55.2)	291 (58.0)	1(ref)		1(ref)	
AG	224 (44.8)	211 (42.0)	1.119 (0.872-1.437)	0.377	1.131 (0.86-1.472)	0.36

Adjusted *P* value calculated by logistic regression with adjustment for age, pre-BMI, SBP, DBP and parity, bold values indicate the P ≤ 0.05.

#### 3.2.2 Stratified analysis results

Subsequently, the associations between two SNPs and susceptibility to GDM in six models were tested using stratified analysis for age or pre-BMI. Notably, protective roles were detected in subjects aged ≥ 30 years for rs13266634 under the dominant model (TT+CT vs. CC: OR = 0.648; 95% CI: 0.431-0.975; *P* = 0.037), codominant homozygous (TT vs. CC: OR = 0.517; 95% CI: 0.307-0.872; *P* = 0.013) and allele model (T vs. C: OR = 0.728; 95% CI: 0.565-0.938; *P* = 0.014). After adjustments, rs13266634 was significantly associated with lower GDM risk under the recessive model (TT vs. CT+CC: OR = 0.615; 95% CI: 0.392-0.966; *P* = 0.035), codominant homozygous model (TT vs. CC: OR = 0.503; 95% CI: 0.294-0.861; *P* = 0.012) and allele model (T vs. C: OR =0.723; 95% CI: 0.557-0.937; *P* = 0.014) (**Table 4**). Moreover, these associations were more evident in subjects aged ≥ 30 years for rs2466293 under the dominant model (GG+AG vs. AA: OR = 1.445; 95% CI: 1.007-2.073; *P* = 0.045) and allele model (G vs. A: OR = 1.337; 95% CI: 1.024-1.747; *P* = 0.033). After these abovementioned factors were adjusted, rs2466293 was significantly related to higher GDM odds under the dominant model (GG+AG vs. AA: OR = 1.579; 95% CI: 1.086-2.295; *P* = 0.017), codominant heterozygous (AG vs. AA: OR = 1.519; 95% CI: 1.020-2.263; *P* = 0.040), and allele model (G vs. A: OR = 1.399; 95% CI: 1.064-1.839; *P* = 0.016) (**Table 4**). However, no significant associations were found in subjects aged < 30 years (**Supplementary Table 1**). Nevertheless, the results indicated no significant relationship between rs13266634 or rs2466293 and GDM susceptibility in subjects in the pre-BMI stratified analysis.

**Table 4:** The associations between SNPs in SLA30C8 gene and GDM risk in subjects aged ≥ 30 years.

Model	Cases (%)	Controls (%)	Crude OR	Crude *P*	Adjusted OR	Adjusted *P*
	(n = 308)	(n = 198)	(95 % CI)		(95 % CI)	
rs13266634						
Codominant model						
CC	98 (31.8)	46 (23.2)	1(ref)		1(ref)	
CT	156 (50.7)	103 (52.0)	0.711 (0.463-1.093)	0.119	0.736 (0.475-1.141)	0.171
TT	54 (17.5)	49 (24.8)	0.517 (0.307-0.872)	**0.013**	0.503 (0.294-0.861)	**0.012**
Aelle model						
C	352 (57.1)	195 (49.2)	1(ref)		1(ref)	
T	264 (42.9)	201 (50.8)	0.728 (0.565-0.938)	**0.014**	0.723 (0.557-0.937)	**0.014**
Dominant Model						
CC	98 (31.8)	46 (23.2)	1(ref)		1(ref)	
TT+CT	210 (68.2)	152 (76.8)	0.648 (0.431-0.975)	**0.037**	0.661 (0.436-1.003)	0.052
Recessive Model						
CT+CC	254 (82.5)	149 (75.2)	1(ref)		1(ref)	
TT	54 (17.5)	49 (24.8)	0.646 (0.418-1.000)	0.05	0.615 (0.392-0.966)	**0.035**
Overdominant model						
TT+CC	152 (49.4)	95 (48.0)	1(ref)		1(ref)	
CT	156 (50.6)	103 (52.0)	0.947 (0.662-1.353)	0.736	0.988 (0.686-1.425)	0.95
rs2466293						
Codominant model						
AA	120 (39.0)	95 (48.0)	1(ref)		1(ref)	
AG	141 (45.8)	81 (40.9)	1.378 (0.939-2.022)	0.101	1.519 (1.020-2.263)	**0.04**
GG	47 (15.2)	22 (11.1)	1.691 (0.953-3.001)	0.071	1.784 (0.994-3.203)	0.053
Aelle model						
A	381 (61.9)	271 (68.4)	1(ref)		1(ref)	
G	235 (38.1)	125 (31.6)	1.337 (1.024-1.747)	**0.033**	1.399 (1.064-1.839)	**0.016**
Dominant Model						
AA	120 (39.0)	95 (48.0)	1(ref)		1(ref)	
GG+AG	188 (61.0)	103 (52.0)	1.445 (1.007-2.073)	**0.045**	1.579 (1.086-2.295)	**0.017**
Recessive Model						
AG+AA	261 (84.7)	176 (88.9)	1(ref)		1(ref)	
GG	47 (15.3)	22 (11.1)	1.441 (0.839-2.475)	0.184	1.447 (0.834-2.508)	0.189
Overdominant model						
GG+AA	167 (54.2)	117 (59.1)	1(ref)		1(ref)	
AG	141 (45.8)	81 (40.9)	1.220 (0.850-1.750)	0.281	1.323 (0.910-1.923)	0.143

Adjusted *P* value calculated by logistic regression with adjustment for age, pre-BMI and SBP. bold values indicate the P ≤ 0.05.

### 3.3 Haplotype and linkage disequilibrium analyses

**Figure 1:**
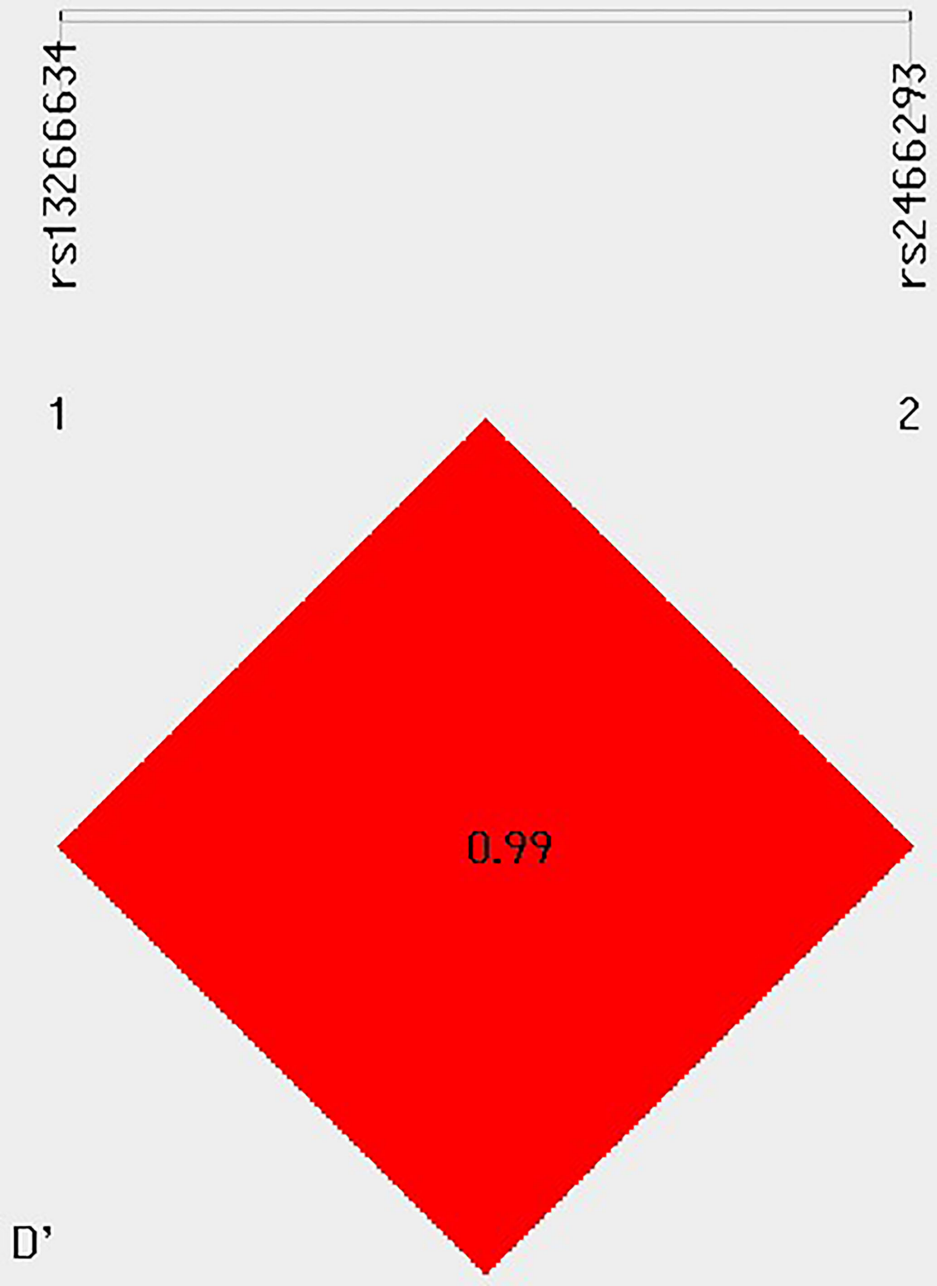
Linkage disequilibrium (LD) between multiple loci of the *SLC30A8* gene (rs13266634 C/T and rs2466293 A/G).

The study found that two SNPs, rs13266634 and rs2466293, were in strong linkage disequilibrium (D′ > 0.99) with each other (**Figure 1**). The CG haplotype consisting of these SNPs was significantly associated with higher GDM risk (OR = 1.231; 95% CI: 1.024-1.48; *P* = 0.026). In addition, the age-stratified analysis revealed that haplotype CG was associated with higher GDM risk in subjects aged ≥ 30 years (OR = 1.328; 95% CI: 1.016-1.734; *P* = 0.037), while haplotype TA was associated with lower GDM risk in subjects aged ≥ 30 years (OR = 0.722; 95% CI: 0.560-0.931; *P* = 0.011). However, no significant associations were found with age < 30 years (**Table 5**).

**Table 5:** Haplotype analysis of the rs132666342 and rs2466293 SNPs of the SLA30C8 gene for the GDM and controls.

Haplotype	Cases (%)	Controls (%)	χ2	*P*	OR (95 % CI)
CA	189 (18.9)	202 (20.1)	0.474	0.49	0.925 (0.741-1.154)
TA	437 (43.7)	475 (47.3)	2.633	0.104	0.864 (0.724-1.03)
CG	373 (37.3)	327 (32.5)	4.932	**0.026**	1.231 (1.024-1.48)
**Haplotype**	**Cases (%)**	**Controls (%)**	**χ2**	***P***	**OR (95 % CI)**
Age (years) <30					
CA	71 (18.4)	132 (21.7)	1.5	0.22	0.817 (0.592-1.128)
CG	139 (36.1)	202 (33.2)	0.922	0.336	1.14 (0.872-1.49)
TA	174 (45.3)	274 (45.0)	0.005	0.939	1.01 (0.781-1.305)
Age (years) ≥ 30					
CA	118 (19.1)	70 (17.6)	0.348	0.554	1.103 (0.795-1.53)
TA	263 (42.6)	201 (50.7)	6.311	**0.011**	0.722 (0.56-0.931)
CG	234 (37.9)	125 (31.5)	4.342	**0.037**	1.328 (1.016-1.734)

### 3.4 The association between polymorphism genotype and blood glucose levels

The fasting glucose and 1-h PG levels of pregnant women with different genotypes were analyzed by age stratification (**Table 6**). The results showed that the glucose indexes of the rs13266634 CC genotype were higher than those of the TT genotype in subjects aged ≥ 30 years (all *P* < 0.05), and the 1-h PG level of the CC genotype was significantly higher than the CT genotype.

**Table 6:** Association between SNPs polymorphisms genotype and blood glucose levels.

Genotype	FPG (mmol/L)	1h-PG (mmol/L)	2h-PG (mmol/L)
rs13266634			
Age (years) < 30			
CC	4.66±0.614	8.49±1.923	7.30±1.925
CT	4.57±0.570	8.38±1.81	7.31±1.594
TT	4.66±1.077	8.55±2.277	7.68±1.925
F	0.903	0.28	2.123
P	> 0.05	> 0.05	> 0.05
Age (years) ≥ 30			
CC	4.79±0.546	9.87±1.717	8.62±1.818
CT	4.74±0.556	9.50±1.884**^b^**	8.25±1.724
TT	4.64±0.482**^a^**	9.10±1.679**^a^**	8.12±1.733**^a^**
F	2.225	5.262	2.766
P	**< 0.05**	**< 0.05**	**< 0.05**
rs2466293			
Age (years) < 30			
AA	4.67±0.887	8.37±2.033	7.48±1.733
AG	4.58±0.606	8.44±1.886	7.27±1.577
GG	4.61±0.493	8.78±1.921	7.49±1.443
F	0.752	1.009	0.945
P	> 0.05	> 0.05	> 0.05
Age (years) ≥ 30			
AA	4.76±0.532	9.40±2.014	8.31±1.917
AG	4.68±0.577	9.62±1.682	8.33±1.693
GG	4.81±0.435	9.56±1.57	8.40±1.478
F	1.739	0.781	0.07
P	> 0.05	> 0.05	> 0.05

^a^LSD was used to compare the blood glucose levels of three rs13266634 genotypes: the difference of blood glucose between CC and TT genotypes was statistically significant, all P < 0.05. ^b^ LSD was used to compare the blood glucose levels of three rs13266634 genotypes: the difference of 1-h blood glucose between CC and CT genotypes was statistically significant, P < 0.05. P < 0.05, bold values indicate the P < 0.05.

### 3.5 Meta−analysis results

**Figure 2:**
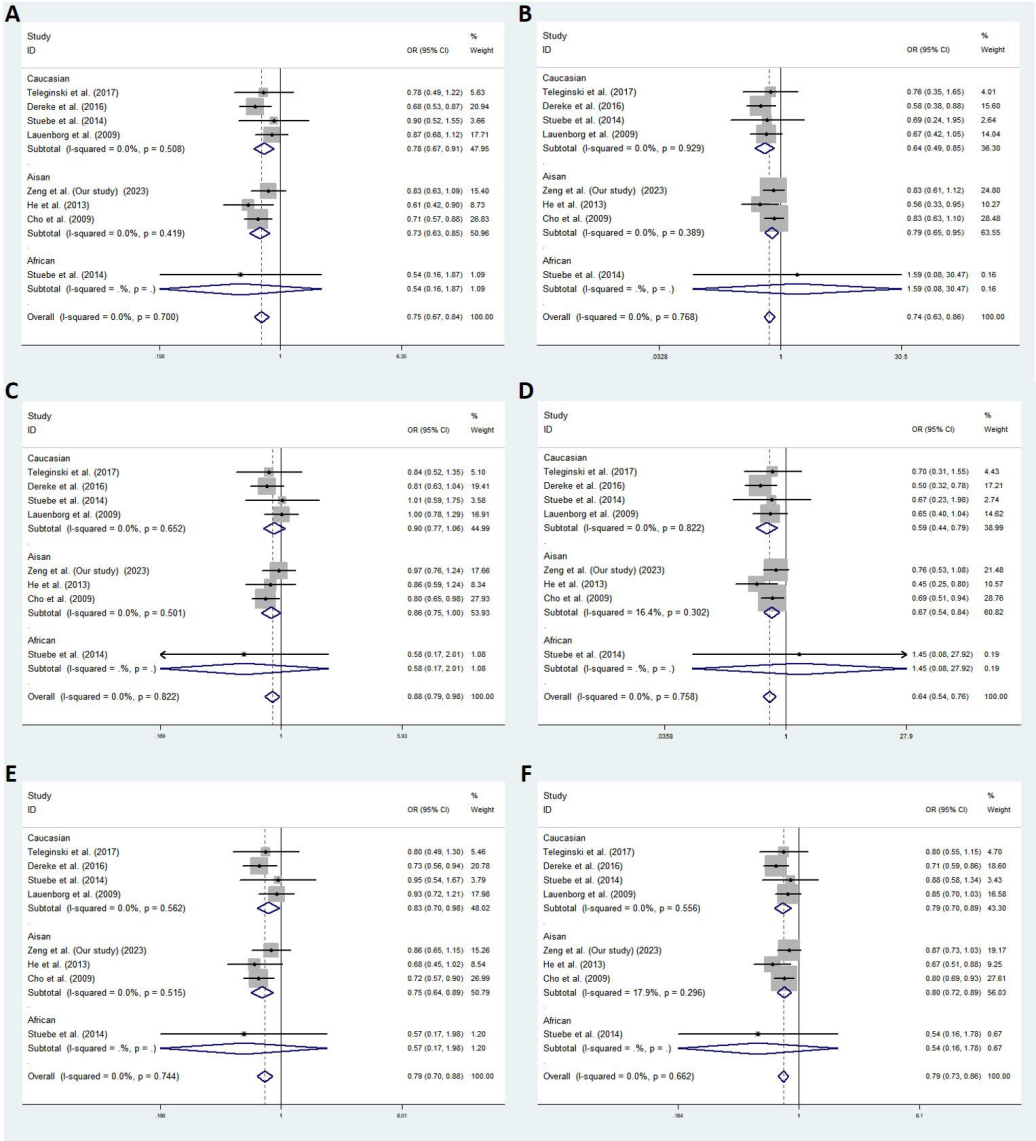
Meta-analysis with a fixed effects model for the association between *SLC30A8* rs13266634 and GDM susceptibility. **(A)** dominant model, TT+CT vs. CC **(B)** recessive model, TT vs. CT+CC **(C)** overdominant model, CT vs. TT +CC **(D)** codominant homozygous model,TT vs.CC **(E)** codominant heterozygous model, CT vs.CC **(F)** allele model, T vs. **(C)** OR: odds ratio, CI: confidence interval, I-squared: measure to quantify the degree of heterogeneity in meta-analyses.

**Figure 3:**
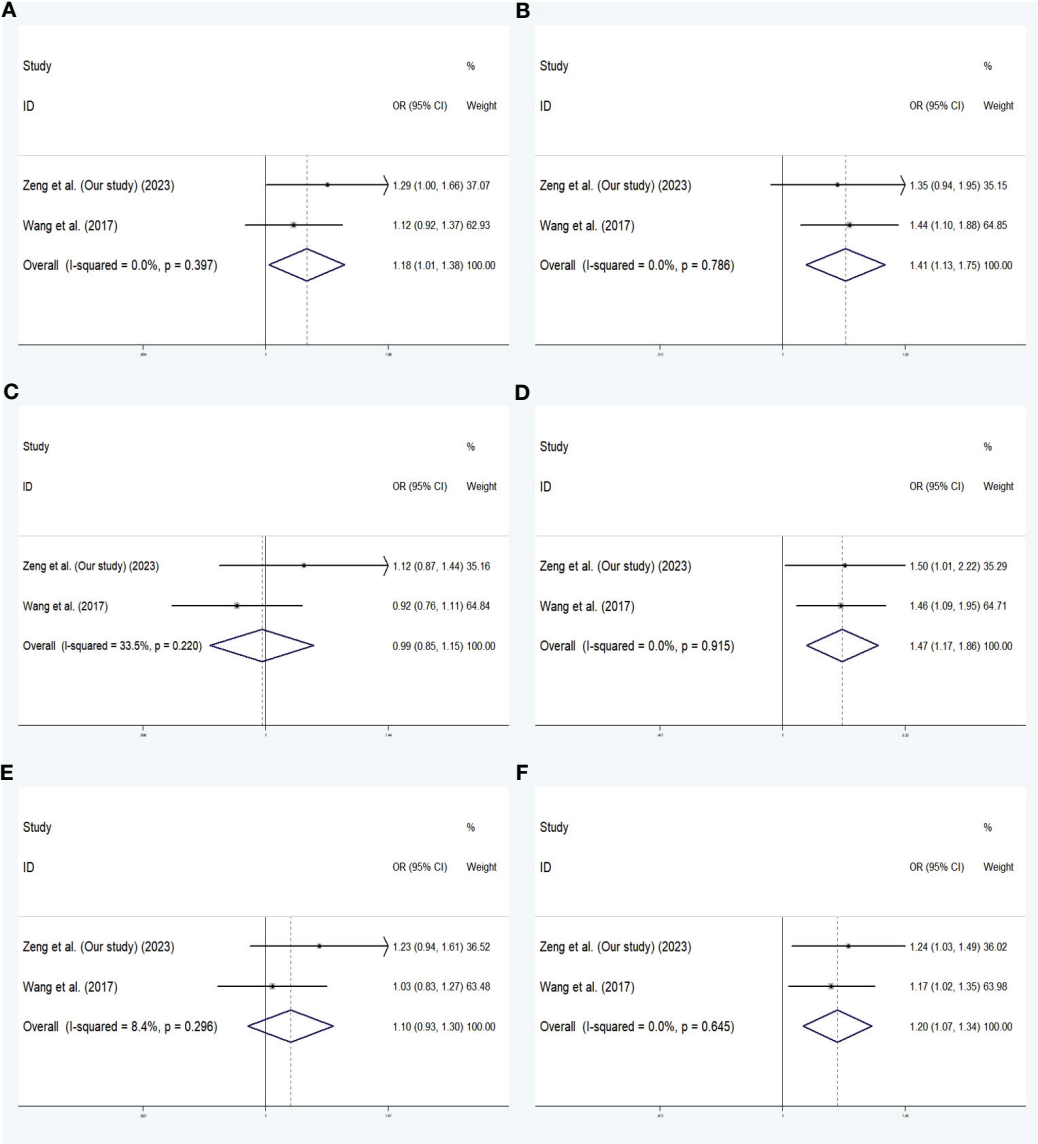
Meta-analysis with a fixed effects model for the association between *SLC30A8* rs2466293 and GDM susceptibility. **(A)** dominant model,GG+AG vs. AA **(B)** recessive model,GG vs. AG + AA **(C)** overdominant model, AG vs. GG +AA **(D)** codominant homozygous model, GG vs. AA **(E)** codominant heterozygous model, AG vs.AA **(F)** allele model, G vs. **(A)** OR: odds ratio, CI: confidence interval, I-squared: measure to quantify the degree of heterogeneity in meta-analyses.

Relevant references were searched for based on the PubMed and Google Scholar databases to evaluate the relationship between *SLC30A8* rs13266634 or rs2466293 and GDM. Eight eligible studies were included in the rs13266634 and GDM analysis, and two studies were related to *SLC30A8* rs2466293 and GDM. In total, the fixed-effects model was used for analysis. Rs13266634 was shown to be significantly associated with a decreased risk of GDM in the following models: dominant model (TT+CT vs. CC: OR = 0.751; 95% CI: 0.674-0.838; *P* < 0.001), recessive model (TT vs. CT+CC: OR = 0.736; 95% CI: 0.629-0.861; *P* < 0.001), overdominant model (CT vs. TT+CC: OR = 0.878; 95% CI: 0.789-0.977; *P* < 0.001), codominant homozygous model (TT vs. CC: OR = 0.643; 95% CI: 0.542-0.763; *P* < 0.001), codominant heterozygous model (CT vs. CC: OR = 0.789; 95% CI: 0.703-0.885; *P* < 0.001), and allele model (T vs. C: OR = 0.795; 95% CI: 0.734-0.860; *P* < 0.001) (**Figure 2**). In addition, *SLC30A8* rs2466293 was associated with increased GDM risk in the dominant model (GG+AG vs. AA: OR = 1.184; 95% CI: 1.013-1.383; *P* = 0.034), recessive model (GG vs. AG + AA : OR = 1.408; 95% CI: 1.135-1.747; *P* = 0.002), codominant homozygous model (GG vs. AA : OR = 1.474; 95% CI: 1.167-1.861; *P* = 0.001), and allele model (G vs. A: OR = 1.195; 95% CI: 1.069-1.336; *P* = 0.002), and no significant association was found in other genetic models (**Figure 3**). There was no obvious evidence of publication bias in the genetic models, and these results are consistent with Egger’s tests (all *P* > 0.05).

## 4 Discussion

The role of genetic factors in the GDM process has been verified by previous findings (12). Rs13266634 is a non-synonymous SNP in *SLC30A8*, and a protective role for the rs13266634 T allele, which reduces GDM risk, has been proposed in a Swedish population (8). In contrast, six studies from Brazil, the United States, Denmark, the Republic of Korea, and China failed to replicate the results (13–18). Therefore, further verification is necessary. Moreover, rs2466293 is a polymorphism in miRNA-binding sites (miR-binding SNP). Recent findings indicated that rs2466293 impacted the development of GDM (19), but more extensive research is needed for verification. This research paper conducted a case-control study to estimate the association of *SLC30A8* rs13266634 or rs2466293 with GDM among six different genetic models in a Chinese population.

In the research process, we explored the relationship between *SLC30A8* gene multiformity and GDM risk. In the overall analysis, the findings indicated that *SLC30A8* rs13266634 showed no association with GDM risk, but *SLC30A8* rs2466293 was shown to be significantly related to increased GDM risk under the dominant (GG+AG), codominant homozygous (GG), and allele(G) genetic models that were unadjusted and adjusted for age, pre-BMI, SBP, DBP, and parity. In this study, women with GDM were older than healthy controls. It has been pointed out that the prevalence of GDM increases with age, and the incidence is higher among women over 30 years of age (20). Therefore, further studies used a cutoff point of 30 years of age and analyzed the association between polymorphic variants and GDM after stratification by age. Interestingly, after adjusting for age, pre-pregnancy BMI, and SBP, our findings indicated that the SNP rs13266634 in *SLC30A8* was found to have a protective effect against GDM risk in subjects aged ≥30 years under the recessive and homozygous dominant genetic models, while *SLC30A8* rs2466293 was significantly associated with increased GDM risk in patients aged ≥30 years under the dominant and heterozygous dominant genetic models. These results are in accordance with some scholarly studies (8, 13, 19). Furthermore, the CG haplotype, comprised of SNPs rs13266634 and rs2466293, was significantly associated with an increased risk of GDM in the overall analysis. In a further analysis stratified by age, the CG haplotype was also associated with an increased risk of GDM in individuals aged ≥ 30 years, while the TA haplotype was associated with a reduced risk of GDM in the same age group. These results suggest that the T allele of rs13266634 in *SLC30A8* can be considered a protective factor for GDM, while the G allele of rs2466293 may be a risk factor for GDM.

Wang et al. found that the C allele of rs2466293 increased susceptibility to GDM in the Chinese population (19), which was consistent with our research findings. In addition, our study found that the TT homozygous genotype of rs13266634 and the T allele decreased the risk of developing GDM in subjects aged ≥ 30 years. Similarly, previous research has demonstrated that the T allele of rs13266634 protects against the risk of GDM in the Swedish population (8), which was consistent with our findings. Moreover, in populations of Filipinos, Swedes, Koreans, and Chinese individuals, there was evidence of an association between the C allele of *SLC30A8* rs13266634 and a higher risk of GDM (8, 13, 14, 18). However, other studies have not found any association between rs13266634 and the risk of GDM in populations of Danes and Europeans (15–17). Inconsistencies in these results may be related to differences in ethnicity, environment, or limited study sample sizes. Therefore, a comprehensive meta-analysis was carried out with a larger number of different populations (ethnicities) to identify the relationship of SLC30A8 SNPs with GDM risk. Rs13266634 was demonstrated to have a protective effect in every genetic model (*P* < 0.05) in eight eligible studies (including our study), and significant findings of rs13266634 could also be observed in both the Caucasian and Asian subgroups. SLC30A8 rs2466293 was found to be significantly related to higher GDM risk in the relevant models (codominant homozygous and allele models) (*P* < 0.05) based on two Chinese population studies.

GDM and T2DM are considered to have similar pathogenesis. In a study of diabetic mice, *SLC30A8* gene expression levels were inhibited in the pancreas of animals with this pathology, indicating that it is related to diabetes (9). Studies have shown that the *SLC30A8* rs13266634 C allele is associated with glucose regulation in GWASs (21, 22). In addition, studies based on fluorescence and radiation have proposed a hypothesis that the rs13266664-T allele reduces *SLC30A8* activity, which changes insulin synthesis and reduces GDM susceptibility based on this mechanism (23–25). In addition, genetic variation in the 3’UTR, a miRNA target gene, can affect the interaction between miRNA and target mRNA. We queried the rs2466293 polymorphism located using the “MirSNP” database (http://bioinfo.life.hust.edu.cn/miRNASNP/). According to the results, it can be inferred that rs2466293 creates eight and destroys three putative miRNA target sites, which may impact the expression of *SLC30A8* and lead to a higher risk of GDM. However, functional research is necessary to further confirm its mechanism.

According to the abovementioned research, this study obtained a conclusion that the age and pre-BMI of the GDM group were significantly higher than those of the control group, and logistic regression analysis indicated that the increase in age and pre-BMI were important risk factors for GDM. SBP, DBP, and parity in the GDM group were significantly higher than those in the other group. It can be inferred that patients with GDM were prone to pregnancy-induced hypertension syndrome. Moreover, a previous study found that the *SLC30A8* rs13266634 C allele was correlated with higher fasting glucose levels among women with gestational high BMI (26). Our study also showed that the *SLC30A8* rs13266634 C allele had an influence on higher fasting glucose, 1-h, and 2-h glucose levels among pregnant women over the age of 30 years, which was similar to the results of previous studies. The *SLC30A8* rs13266634 C allele may affect the normal secretion of insulin. Wang et al. found a significant relationship between the C allele of rs2466293 with higher plasma glucose (19), but no differences were found in our study. Therefore, further relevant research is necessary.

There are still several limitations in this study. First, due to the modest sample size of the GDM and control groups, future studies need to validate our observations in a larger cohort. Second, the data used in this study were insufficient, such as the lack of fasting insulin data, to accurately measure and evaluate pancreatic islet β-cell function. Finally, the study subjects were limited to Chinese individuals, and additional research is necessary to confirm our findings in diverse populations.

## 5 Conclusions

In conclusion, in subjects aged ≥ 30 years, *SLC30A8* rs13266634 exhibited a protective relationship against GDM susceptibility, while the results indicated associations of rs2466293 with the risk of GDM. The haplotype CG was also associated with a higher risk of GDM, and the haplotype TA was associated with a lower risk of GDM in subjects aged ≥ 30 years. In general, our findings provide more clues for studying the precise mechanism of the development of GDM.

## Data availability statement

The original contributions presented in the study are publicly available. This data can be found here: PRJEB61053, ERZ16808993.

## Ethics statement

The study was agreed by the Ethics Committee of Shunde Maternal and Child Health Hospital of Guangdong Medical University. The patients/participants provided their written informed consent to participate in this study.

## Author contributions

QZ, BT and FH contributed equally to this study. QZ, FH and BT collected clinical data and samples. QZ, XH and JH performed data analyses. QZ, YW and RG wrote the manuscript. JH and YW supervised the whole research. All authors contributed to the article and approved the submitted version.
